# Retrospective study on bovine clinical mastitis and associated milk loss during the month of its peak occurrence at the National Dairy Farm in the Emirate of Abu Dhabi, United Arab Emirates

**DOI:** 10.3389/fvets.2022.1070051

**Published:** 2022-12-20

**Authors:** Gobena Ameni, Berecha Bayissa, Aboma Zewude, Berhanu Adenew Degefa, Khaja Mohteshamuddin, Gopala Kalaiah, Meera Saeed Alkalbani, Yassir Mohammed Eltahir, Mohamed Elfatih Hamad, Markos Tibbo

**Affiliations:** ^1^Department of Veterinary Medicine, College of Food and Agriculture, United Arab Emirates University, Al Ain, United Arab Emirates; ^2^Aklilu Lemma Institute of Pathobiology, Addis Ababa University, Addis Ababa, Ethiopia; ^3^Vaccine Production and Drug Formulation Directorate, National Veterinary Institute, Bishoftu, Ethiopia; ^4^Department of Integrative Agriculture, College of Food and Agriculture, United Arab Emirates University, Al Ain, United Arab Emirates; ^5^National Dairy Farm, Al Ain, United Arab Emirates; ^6^Animal Health Division, Abu Dhabi Agriculture and Food Safety Authority, Abu Dhabi, United Arab Emirates; ^7^Subregional Office for the Gulf-Cooperation Council States and Yemen, Food and Agriculture Organization of the United Nations, Abu Dhabi, United Arab Emirates

**Keywords:** clinical mastitis, milk loss, incidence rate, dairy cattle, United Arab Emirates

## Abstract

**Background:**

Commercial dairy establishments are relatively young in the United Arab Emirates (UAE), and as a result, there is lack of epidemiological data on mastitis in dairy farms.

**Methods:**

A retrospective data of seven years (2015–2021) were used to estimate the cumulative average monthly incidence rate of bovine clinical mastitis and evaluate associated milk loss at the National Dairy Farm. Data were extracted from the records of lactating dairy cows (*n* = 1300–1450) and analyzed using repeated measure and one-way ANOVA, non-parametric Spearman correlation, paired and unpaired *t* tests.

**Results:**

The highest average cumulative monthly incidence rate was 49 cases per 1000 cows-year that was recorded in 2019 while the lowest was 19 cases per 1000 cows-year in 2021. The cumulative average monthly incidence rate of clinical mastitis significantly (*p* < 0.001) varied among the seven years. The cumulative average monthly incidence rate was associated with average monthly humidity (*p* < 0.01) and average monthly rainfall (*p* < 0.05); however, it was not associated with the average monthly temperature (*p* > 0.05). The average daily milk yield of cows with clinical mastitis (Mean ± SEM; 18.6 ± 0.54 kg) was significantly (*p* < 0.001) lower than the average daily milk yield of clinical mastitis free cows (40.5 ± 0.29 kg). The largest average monthly milk loss due to clinical mastitis was 5% of the average total monthly milk production in 2019 while the lowest was 2% of the average total monthly milk production in 2021.

**Conclusion:**

The result of the study indicated the direct influence of weather conditions such as increased rainfall and humidity, which caused an upsurge in the incidence rate of clinical mastitis, leading to an increased loss in milk and hence the economy of the dairy farm. Proactive preventive measures along with good dairy farm practices that help mitigate the impacts of harsh weather conditions are recommended.

## Introduction

Bovine mastitis is defined as an inflammation of the mammary gland that can be either caused by infectious agents or non-infectious agents. According to the review published recently, bovine mastitis can be classified into three classes based on the degree of inflammation, namely clinical, sub-clinical, and chronic mastitis (1). Clinical mastitis is characterized by visible abnormalities of the udder and or the milk. However, sub-clinical mastitis does not show visible abnormality in the udder or milk, but milk production decreases with an increase in the somatic cell count (SCC) (2). On the other hand, chronic mastitis is characterized by a persistent inflammation of the mammary gland in dairy animals.

Based on the etiologic bacteria, mastitis is further classified into contagious and environmental mastitis. As it can witnessed from the literature, the most common bacteria, which because contagious mastitis are *Staphylococcus aureus* and *Streptococcus agalactiae* though *Mycoplasma bovis* and Corynebacterium species can also cause contagious mastitis (1, 3). Contagious mastitis causing bacteria live on the udder and teat skin from where they can easily colonize the teat canal and infect the udder. Contagious pathogens are the common causes of sub-clinical mastitis leading to elevated SCC and deterioration of the quality of the milk (4, 5). On the other hand, environmental mastitis is caused by pathogens which live in the cow's environment including in the bedding and housing of the cows waiting for a chance to cause infection (1). Environmental pathogens are extremely heterogenous and the most frequently encountered species are Streptococci species (except *Streptococcus agalactiae*) and coliform species, and are considered to be common causes clinical mastitis (3).

In spite of continuous efforts exerted to control bovine mastitis for the last several years, the disease remains as a bottleneck for the dairy development because of its huge economic loss in dairy cows resulting in a reduction of milk production, loss in milk quality and quantity, losses due to discarded milk, premature culling, treatment costs, and extra labor cost (6). The financial loss caused by mastitis were reported by various authors from different countries, and for instance, a total economic loss of US$ 2 billion was recorded in the USA in 2009 due to mastitis (7). Furthermore, an estimated annual economic loss of US$ 98,228 million was reported from India due to mastitis (8). Additionally, according to the review published earlier (3), the UK dairy industry loses £168 million annually due to clinical mastitis. Discarded milk and lowered milk production accounting for about 80% of the total cost associated with mastitis (9). In addition, annual mortality rate of 0.6% was reported in lactating cows due to clinical mastitis (10). On top of these, mastitis has significant public health implication because of the extensive use of antibiotics for the treatment of mastitis in dairy cattle that increases the exposure of humans to antibiotic resistant strains of bacteria through the food chain (11). The potential spread of zoonotic organisms through milk remains a risk to the public especially in regions where unpasteurized milk and its products are consumed (3). Thus, the financial implications of mastitis and its the public health significance require serious attention at herd, ministry and country levels.

The UAE Government has set a goal to become one of the countries with the best performance in food security index with the target of becoming one of the top 10 countries in food security index by 2051 (12). It is believed that dairy production is one of the key contributors to the food security agenda of the country, as it serves as a source of fresh milk and dairy products. Despite the challenges of climatic conditions and feed shortage from domestic supply, the dairy sector in the UAE is growing. Dairy products' market in the UAE was estimated at US$1.66 billion in 2020 and has been forecasted to reach US$2.47 billion by 2026 (13). The drivers for the rise in dairy products' market in the UAE include consumer preference for organic milk, the growing demand of young population for dairy products, and the penetration of international players. The growing demand for dairy products and the growth of market of dairy products are attracting dairy farm investment and thereby contributing to the establishment of dairy farms. There are good number of large-scale dairy farms in the UAE, which play leading roles as sources of milk and dairy products in the country.

However, although the occurrence and spread of clinical mastitis in these dairy farms are likely in connection to the growth in dairy production, there is shortage of published scientific data on the magnitude of clinical mastitis in the dairy farms in the UAE. The present study was conducted to estimate the incidence rate of clinical mastitis and associated milk losses at the National Dairy Farm that is located in the Al Ain region of the UAE.

## Materials and methods

### Study farm and its settings

**Figure 1:**
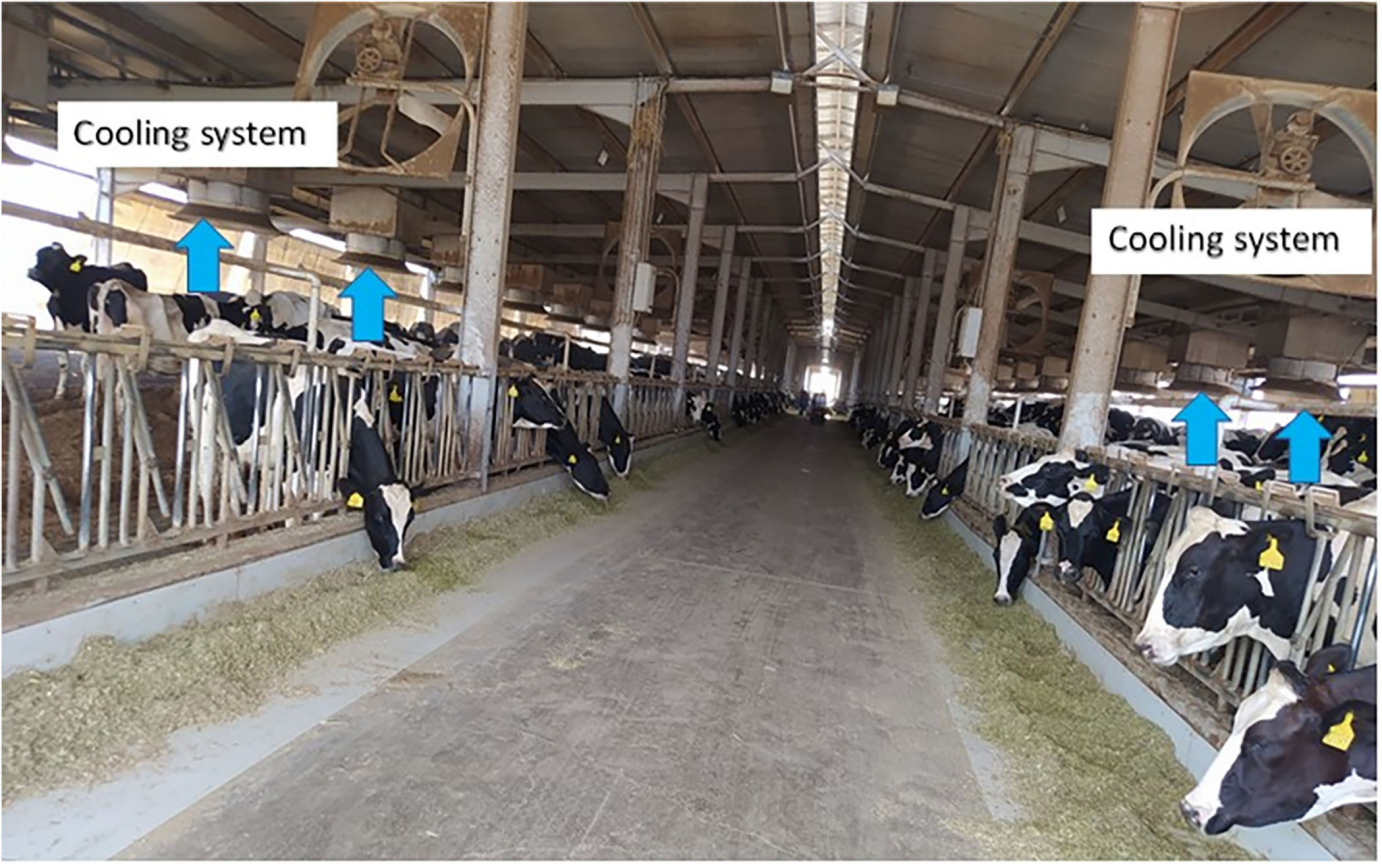
One of the barns of the National Dairy Farm. The barns are open with shades while the floor is sandy. The arrows indicate the in-built cooling system. Most of the ingredients of the feed including hay are imported from other countries.

Emirates Food Industries (EFI) LLC currently owns two dairy farms near Al Ain with estimated dairy cattle of over 5,700 heads. One of the farms is the Masakin Dairy farm LLC that started operation in 1997 and grew to its current size of a total Holstein Friesian herd of over 1,600 heads, consisting of a milking herd of 850 cows plus dry and replacement stock. The second farm is the National Dairy Farm LLC, which started operation in 1999 and grew to its current size of a total Holstein Friesian herd of over 3,800 head, consisting of a milking herd of over 1,450 milking cows plus dry and replacement stock. Both farms are located 30 km outside Al Ain City on the Al Ain—Dubai road. The cows are housed under purpose-built facilities with a patented cooling system that ensures the environment an ambient temperature of no higher than 20°C even if the outside temperature is very high (Figure 1).

Both farms are managed together on a commercial basis producing ~38 million liters in 2021 of grade one raw milk, as well as livestock including Angus & Charolais cross calves and manure sales, respectively, for meat and fertilizer, mainly for UAE market. The cows are milked four times daily for their comfort through a modern facility using best practices and latest technology including a system of daily monitoring of individual cow somatic cell counts (SCC). Both farms can be benchmarked favorably over any global dairy enterprise and continue to improve in all aspects. The farms use their own in-house laboratory to monitor milk quality and herd health status. The farms operate Dairycomp 305 Herd Management software and SAP ERP system. Both farms hold Grade A certificates issued by Abu Dhabi Agriculture and Food Safety Authority (ADAFSA), ISO 9001:2015 Quality Management System Certification and ISO 22000:2018 Food Safety Management System Certification. The dairy farms anticipate to achieve ISO 14001:2015 Environmental Management Certification.

Feed is the highest expensive item and the management works with the best global dairy nutritionists to ensure maximum efficiency. Feed is sourced globally depending on quality, pricing, and availability in conjunction with a sister company—National Feed Company. Milking herd is fed TMR four times daily and routinely analyzed using NIR technology. The total workforce for both farms is a team just under 100 people but this covers all aspects of the business. The dairy farms are considered amongst the best in the UAE and work closely with the UAE University from practical training aspect to research work along with an extensive global network of institutions. The business has continued to grow by matching its market demand from 16 million liters in 2009 to over 38 million liters in 2022. Further market-led expansion is planned as their new processing facility of the National Dairy comes on line soon.

### Examination of udder and milk for the diagnosis of clinical mastitis

The definition of the clinical mastitis was based on the observation of abnormalities in the udder, milk or in both. In addition, in severe cases, additional abnormalities of body systems of cows can occur. According to the protocol of the Farm, the fore streams of milk were collected and examined visually on routine basis for any abnormalities in the milk immediately before the routine milking. Thus, mastitis cases were considered as mild (grade I) when changes were observed only in the milk including presence of flaks, clots, blood, watery consistency while the udders and the appetites of the cows were normal. On the other hand, cows with visible changes in the milk and with swollen udder and with normal appetite were classified as moderate (grade II) cases. Lastly, severe (grade III) mastitic cows were cows with visible changes in the milk, swollen udders and with additional clinical signs such as loss of appetite. Besides, the Farm has installed Saber™ Somatic cell counter (SCC) (registered in New Zealand) is attached to the milking line and SCC is monitored by color alerts that are displayed by the counter. All cows with red display (SCC >800,000 cells/mL of milk) are moved to Medics Pen for treatment and follow up and the bulk tank milk sample is checked for SCC on periodically, and it is always <200,000 cells/ml.

### Study cows and extraction of data from their records

The records of all lactating dairy cows (the number varied from 1,300 to 1,482) kept by the Farm during the 7 years (2015–2021) were used for data extraction. The data were extracted retrospectively on clinical mastitis and milk production from the records of lactating dairy cows. The records of cows with missing data were excluded from the analysis. The outcome variable was incidence rate of clinical mastitis. The number of cases were recorded on a monthly basis for the 7 years. For the estimation of the cumulative monthly incidence rate of clinical mastitis, the recorded monthly data of clinical mastitis were used. Meteorological data on the average monthly temperature, relative humidity, and rainfall of the Farm were obtained from the National Center of Metrology of the UAE (http://www.ncm.ae). The incidence of clinical mastitis was considered as the predictor milk production. Data were collected on the milk production by clinical mastitis free cows, and clinical mastitis positive cows. The ratio of clinical mastitis positive cows to clinical mastitis free cows varies from 1:17 to 1:82 during the 7 years of study period. The amount of discarded milk was extracted from the records, and used for the estimation of the milk yield of cows with clinical mastitis. The average daily milk yield of a cow with clinical mastitis was calculated by dividing the average monthly produced medic milk (milk produced by cows with clinical mastitis) for the monthly average number of clinical mastitis cases. Similarly, the daily milk yield of clinical mastitis free cow was calculated by dividing the average monthly produced milk by clinical mastitis free cows to the average monthly number of clinical mastitis free cows. Reduction of the average daily milk yield due to clinical mastitis was considered to be the difference between the average daily milk yield of clinical mastitis free cows and the average daily milk yield of cows with clinical mastitis.

### Evaluation of the data

The data on clinical mastitis were collected by an experienced veterinarian working for the Farm. The veterinarian used presence of abnormalities either in the udder, in the milk or in both the udder and milk for classifying cows as clinical mastitis cases or as free from clinical mastitis. These criteria are also well-recognized in the literature and can fulfill the definition of clinical mastitis. But the veterinarian did not do further confirmation of the clinical mastitis cases with bacteriological examination of the milk and identification of the bacteria that caused clinical mastitis in the study Farm. Nonetheless, the clinical examination of udder and milk for the detection of clinical mastitis could be reliable and valid procedures, and hence the data could be considered as reliable and acceptable. Regarding the milk production, the reliability and validity of the data were checked by searching of the error in the data set. In addition, it was learned that the data recording system of the Farm is well-organized and operates using Dairycomp 305 Herd Management software and SAP ERP system.

### Data management and statistical analysis

Statistical analysis was made and graphs were generated using Graphpda Prism 8. Descriptive analysis was conducted using mean and standard errors of mean (SEM) while data that failed the normality test such as monthly rainfall were summarized using median and interquartile range (IQR). The association of average cumulative monthly incidence rate of clinical mastitis with weather conditions was done using Pearson's correlation coefficient. The monthly average milk loss due to clinical mastitis cows was summarized using mean and 95% confidence intervals (CI). The discarded milk and reduction in milk yield due to clinical mastitis were compared using paired *t*-test. The monthly average daily milk yield of mastitis free and mastitis cows was compared using paired *t*-test. In addition, *t*-test with Welch's correction (unequal sample size) was used for analysis the difference in mean cumulative incidence of clinical mastitis between the hot and cold seasons.

Over the seven study years, monthly average milk production of a farm, monthly average daily milk yield reduction of a mastitis cow, and monthly average discarded milk and monthly cumulative incidence were analyzed by repeated measures ANOVA with Geisser-Greenhouse with Tukey's multiple comparison test. In all cases, a 95% confidence level and a significance level of 5% were used to define statistical significance.

## Results

### Cumulative incidence of bovine clinical mastitis for 7 years at the National Dairy Farm

**Figure 2:**
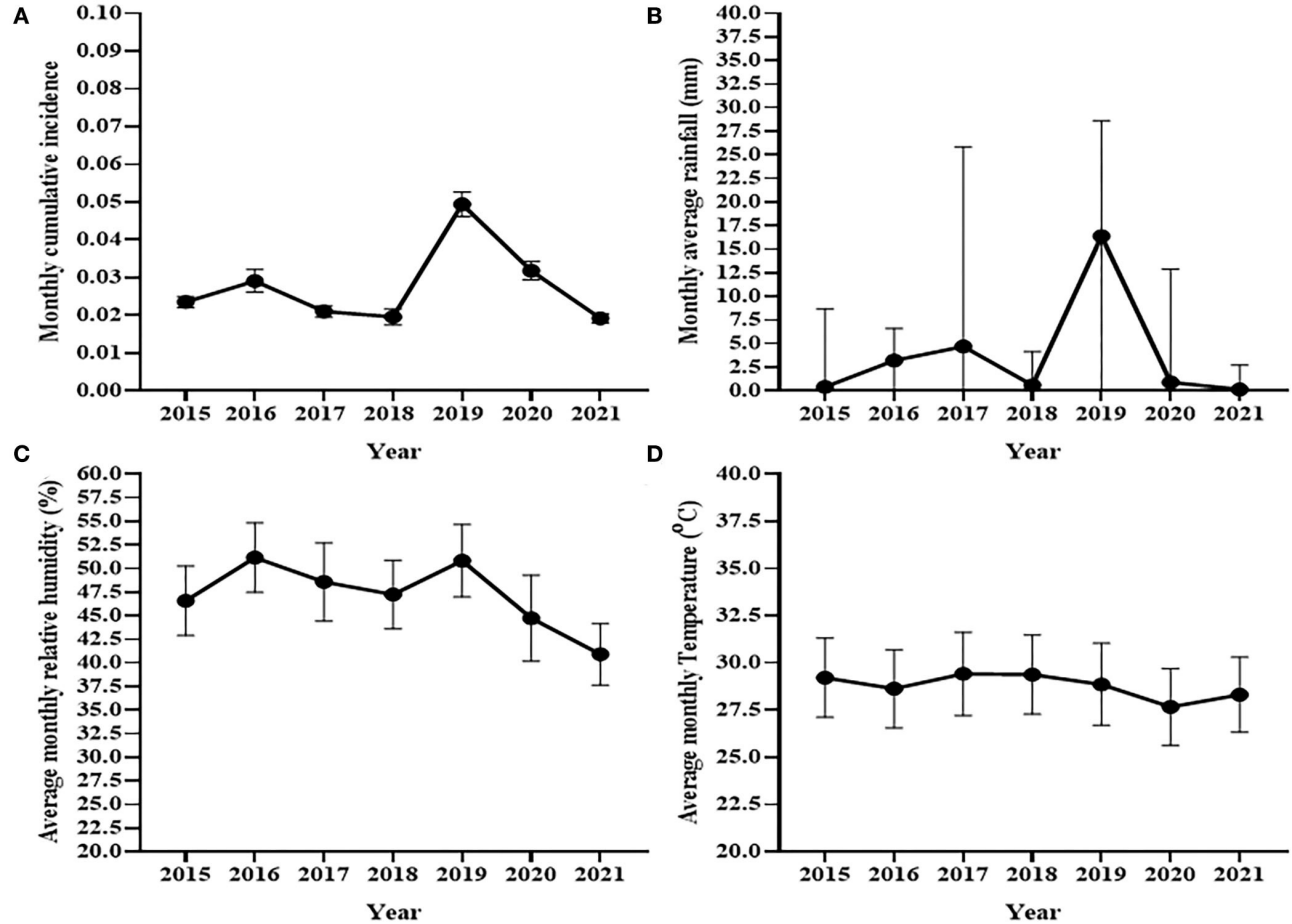
Trends of cumulative incidence of clinical mastitis at the National Dairy Farm **(A)** and trend of monthly rainfall **(B)**, average monthly relative humidity **(C)**, and average monthly temperature **(D)** during seven study years. The trend of observed monthly cumulative incidence of clinical mastitis was similar to the trend pattern of rainfall and relative humidity recoded in the areas while it seems different from trend of monthly average temperature recorded in the area.

Figure 2 shows the trend of the average monthly cumulative incidence of bovine clinical mastitis at the National Dairy Farm and the trends average monthly humidity, temperature and rainfall of the dairy of the area between 2015 and 2021. The average monthly cumulative incidence of bovine clinical mastitis was 49 cases per 1,000 cows at risk of infection in 2019 which was highest while it was 19 cases per 1,000 cows at risk of infection in 2021 that was the lowest average monthly cumulative incidence (Figure 2A). The result of the analysis using repeated measures one-way ANOVA indicated a significant difference [*F*_(2.6,20.8)_ = 24.56, *p* < 0.0001] in average monthly cumulative incidence of clinical mastitis over years. The cumulative incidence of the remaining 5 years laid between the cumulative incidences recorded in 2019 and 2021. The average monthly rainfall varied during the 7 years (Figure 2B). A significant increase in the median monthly rainfall (Median = 16.4 mm, IQR = 0–28.6) was observed in 2019 as compared to the median monthly rainfall recorded for the other years (Figure 2B). Similarly, a rise was observed in the mean monthly average humidity during 2016 and 2019 as compared to other years (Figure 2C) while no variation was observed in the average monthly temperature of a year over seven study years (Figure 2D).

**Figure 3:**
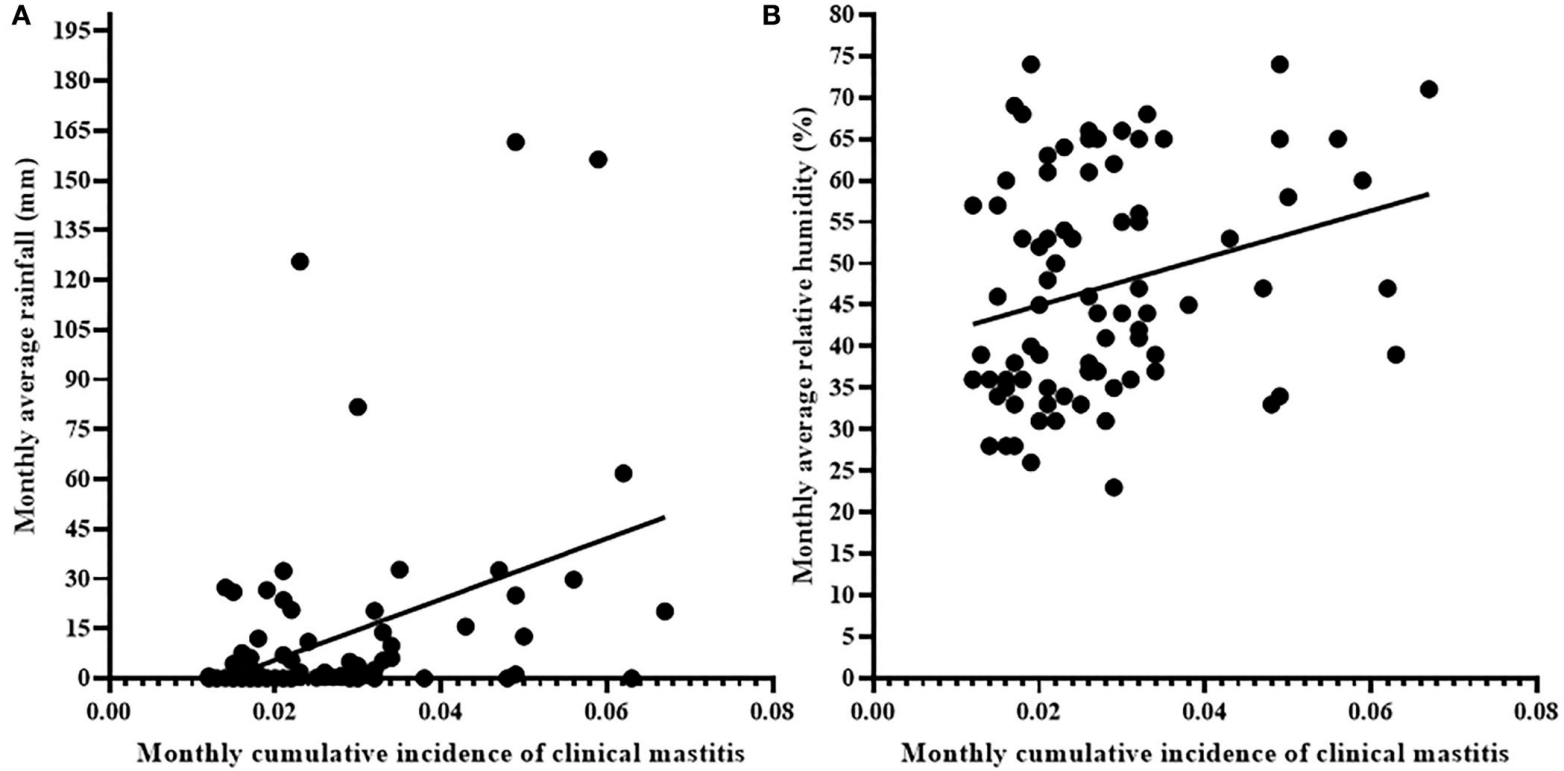
Correlation of monthly cumulative incidence of clinical mastitis with monthly rainfall **(A)** and average monthly relative humidity **(B)**. Solid black circle represents an individual clinical mastitis case. There were association of monthly cumulative incidence of clinical mastitis to monthly rainfall (*r* = 0.252, *p* < 0.05) and average monthly relative humidity (*r* = 0.286, *p* < 0.01) recorded in the area.

Figure 3 shows the association between cumulative incidence rate of clinical mastitis and average monthly rainfall (Figure 3A) and humidity (Figure 3B). The cumulative monthly incidence rate of clinical mastitis was associated with monthly rainfall (*r* = 0.252; 95% CI = 0.033–0.447, *p* < 0.05; Figure 3A) and average monthly humidity (*r* = 0.286, 95% CI = 0.069–0.476; *p* < 0.01; Figure 3B). However, there was no association (*p* > 0.05, *r* = −0.095; 95% CI = −0.309–0.128) between the average monthly temperature and monthly cumulative incidence of clinical mastitis.

**Figure 4:**
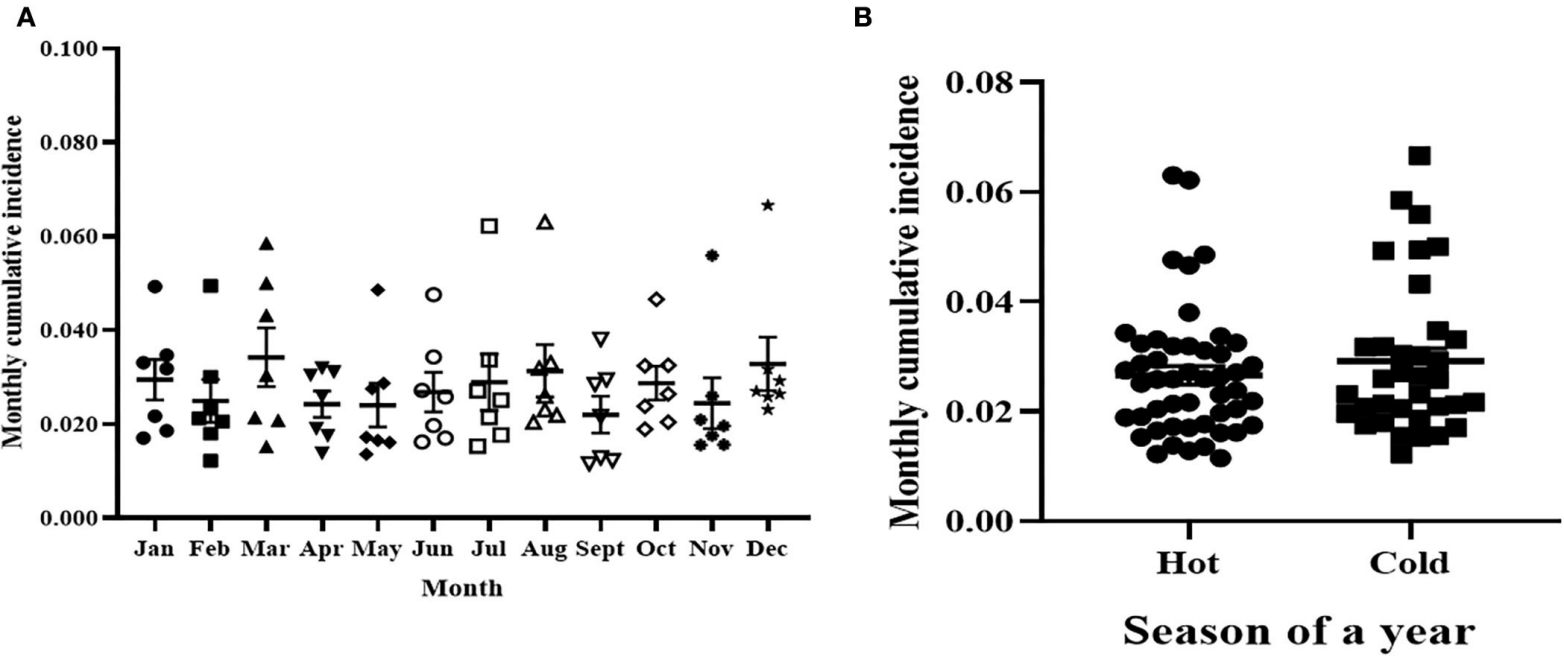
The cumulative incidence of clinical mastitis during the months **(A)** and seasons **(B)** of years. **(A)** The average cumulative incidence of clinical mastitis during the months of the 7 years. The difference in monthly cumulative incidence of clinical mastitis was not statistical significantly. **(B)** The average cumulative incidence of clinical mastitis during the hot and cold seasons of the year. Furthermore, there was no difference (0.027; 95% CI = 0.023–0.030) in average monthly cumulative incidence of clinical mastitis between the hot and relatively cold (0.029; 95% CI = 0.024–0.034) seasons.

Figure 4A shows the variation of cumulative incidence rate clinical mastitis among different months of the 7 years. There were seven observations for each month as presented in the individual value plot. The highest mean monthly cumulative incidence rate of clinical mastitis was 34 per 1,000 susceptible cows and was recorded in March while the lowest mean cumulative incidence was 22 cases per 1,000 susceptible cows that was recorded in September. However, there was no significant [*F*_(2.6,15.7)_ = 1.88, *p* > 0.05] variation of monthly cumulative incidence among the 12 months of the year during the 7 years. Furthermore, the 12 months were grouped into hot (April to October) and relatively cold (November to March) seasons based on the UAE climate condition and the cumulative incidence rates of clinical mastitis were compared between the two seasons (Figure 4B). The mean cumulative incidence of clinical mastitis was 0.027 (95% CI = 0.023–0.030) during the hot season while it was 0.029 (95% CI = 0.024–0.034) during the cold season. Thus, the difference in cumulative incidences of clinical mastitis during hot and cold seasons in the study period was not significant (*t* = 0.9086, *p* = 0.3669).

### Dynamics of milk production at the National Dairy Farm for 7 years

**Figure 5:**
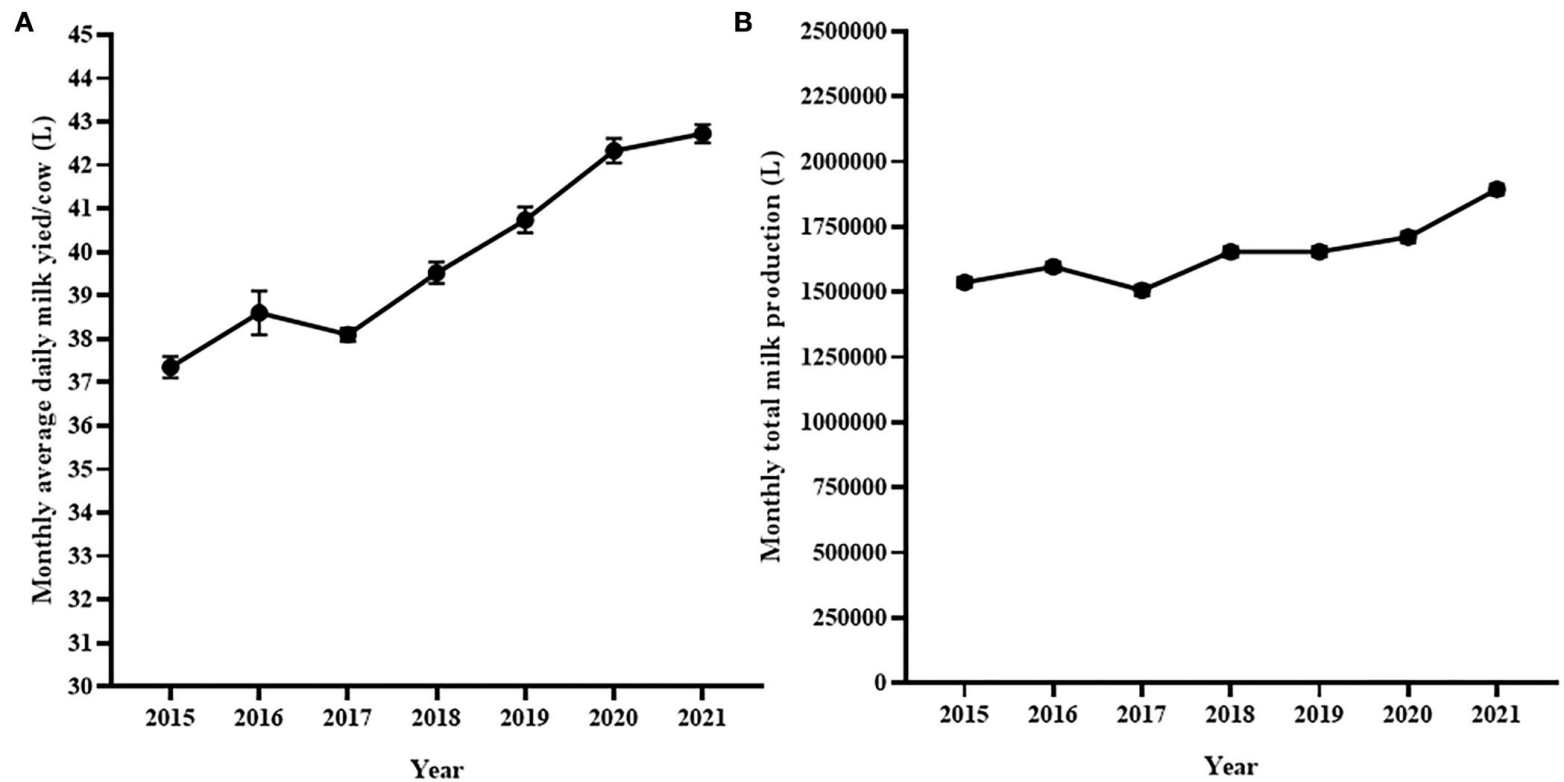
Trend of monthly average daily milk production **(A)** and monthly average total milk production **(B)** for seven study years. Both monthly average daily and monthly total milk production at the National Dairy farm was gradually increasing during the study years except in 2017.

The average monthly number of lactating cows was smallest (*n* = 1300) in 2015 while it was the largest (*n* = 1,450) in 2021. Figure 5 shows monthly average milk yield per cow per day and the average monthly total milk production for 7 years. The average monthly milk yield per day per cow varied significantly [Repeated measure ANOVA with Geisser-Greenhouse's epsilon correction *F*_(2.3,25)_ = 69.47, *p* < 0.0001; Figure 5A] over 7 years. The average monthly milk yield per cow per day was progressively increased during the 7 years except in 2017. The average monthly milk production also showed an increasing pattern during the 7 years (2015–2021) although there was a decrease of milk production in 2017. Overall, a significant difference in average monthly milk production of the different years was observed [Repeated measure ANOVA with Geisser-Greenhouse's epsilon correction *F*_(2.3,24.59)_ = 77.51, *p* < 0.0001; Figure 5B].

### Milk losses due to clinical mastitis and associated economic loss

#### Daily milk yield

**Figure 6:**
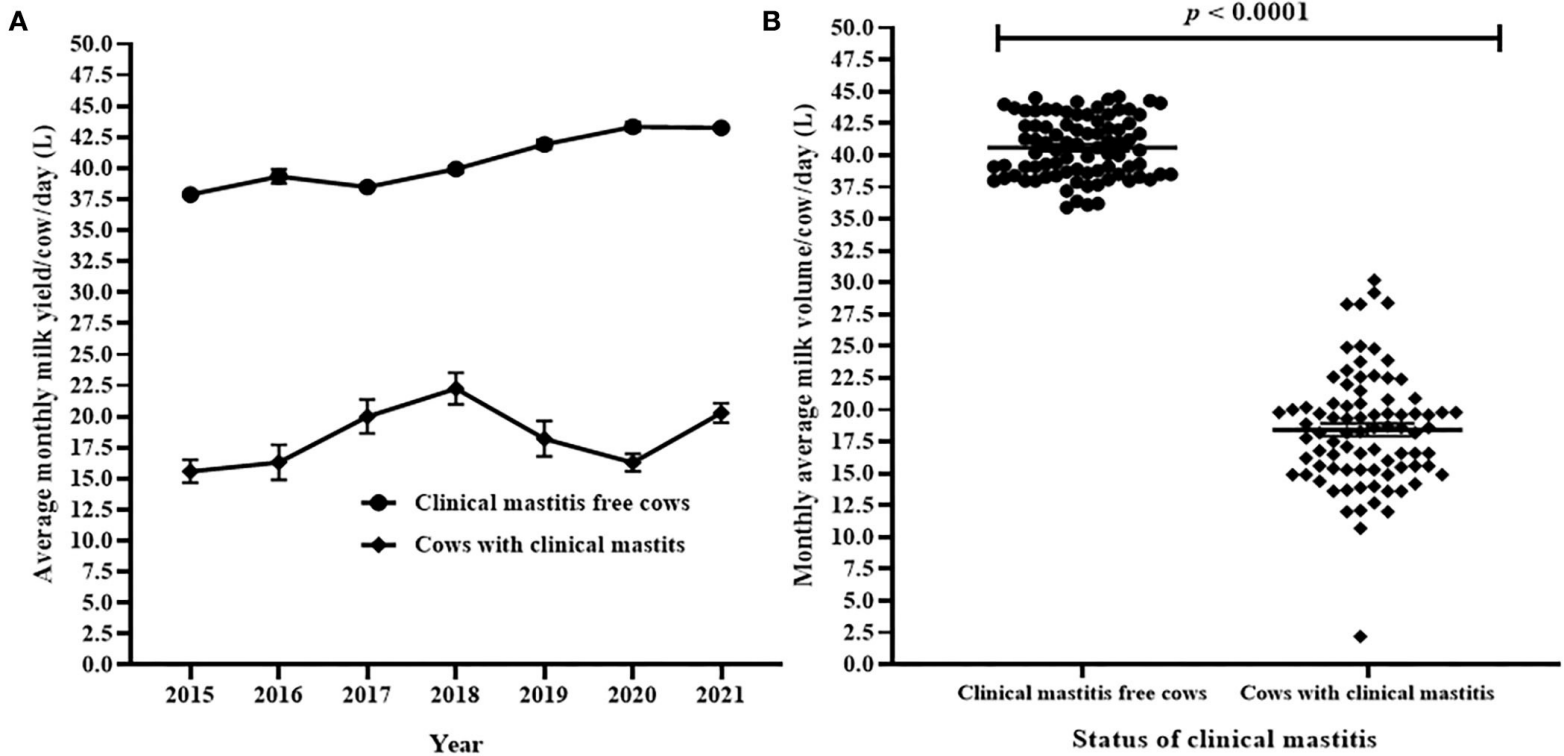
Comparison of average monthly daily milk yield per cows with clinical mastitis and cows free from clinical mastitis. **(A)** The difference of annual average monthly daily milk yield between cows affected by clinical mastitis and cows free from clinical mastitis. In **(B)**, each solid circle and solid square represent the observed average monthly daily milk yield of cows with clinical mastitis and cows free from clinical mastitis, respectively. The recorded difference in the observed average monthly daily milk yield between the groups of cows was significant (Welch's correction *t* = 39.68, *p* < 0.0001).

The average daily milk yield by cows with clinical mastitis and cows free from clinical mastitis are presented in Figure 6A. The trends showed significant differences in average daily milk yield of cows with clinical mastitis and cows free from clinical mastitis during the 7 years (Figure 6A). Cumulatively, the average (Mean ± SEM) of milk production per cow per day in cows affected by clinical mastitis and in cows free from clinical mastitis were compared. The result indicated that the average daily milk production in clinical mastitis free cows (40.6 ± 0.26 kg) was significantly greater (Welch's correction *t* = 39.68, df = 124.9, *p* < 0.0001) than the average daily milk production in cows affected with clinical mastitis (18.4 ± 0.50 kg; Figure 6B). The mean difference of milk yield between cows free from clinical mastitis and cows with clinical mastitis was 22.2 ± 0.55 kg liters.

#### Monthly milk yield

**Figure 7:**
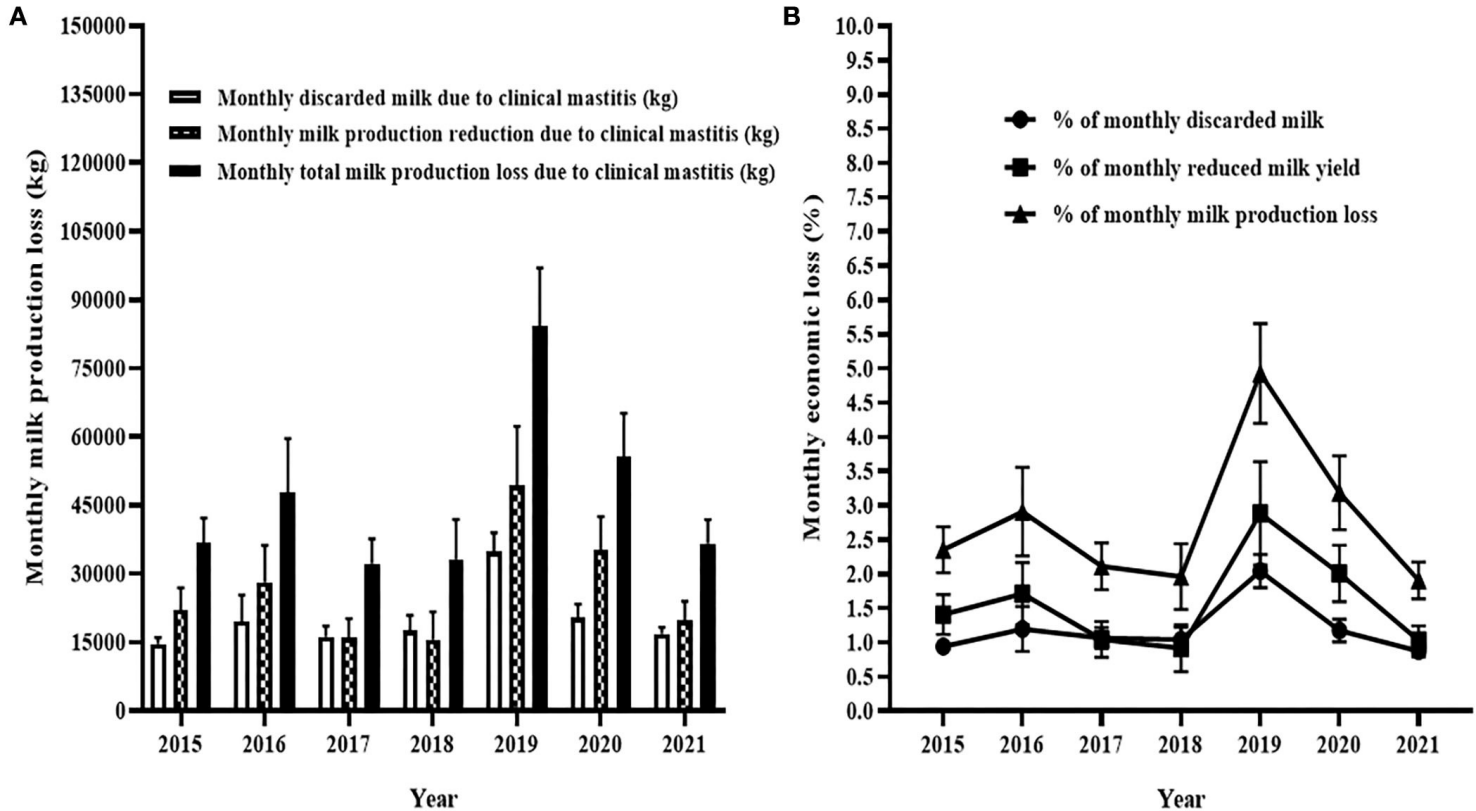
Monthly milk production loss due to clinical mastitis. In **(A)**, unshaded solid bar with error represents mean and 95% CI of monthly discarded milk due to clinical mastitis while half-shaded solid bar with error indicates milk production loss due to reduction in milk yield of cows affected by clinical mastitis and shaded solid bar with error shows to mean of monthly total milk production in related to occurrence of clinical mastitis. Similarly, **(B)** shows the percentage of monthly discard milk, monthly reduction in milk production, and monthly total production loss due to clinical mastitis.

The Figure 7 shows the estimated milk losses due to reduction of milk yield and discarded milk due to clinical mastitis. The average monthly milk loss due to clinical mastitis was < 50,000 kg during the five years (2015–2018 and 2021; Figure 7A) of the 7 years. However, the average monthly milk loss was 82,000 kg during 2019 and 55,000 kg during 2020 (Figure 7A). In terms of percentage, the average monthly milk loss due to clinical mastitis was the least in 2021 which was 1.9% of the average total monthly milk production while it was the largest in 2019 which was 5% of the average total monthly milk production (Figure 7B). The losses were 2% during each of 2017 and 2018 while the losses were 2.4, 3.0, and 3.2% in 2015, 2016, and 2020, respectively.

**Figure 8:**
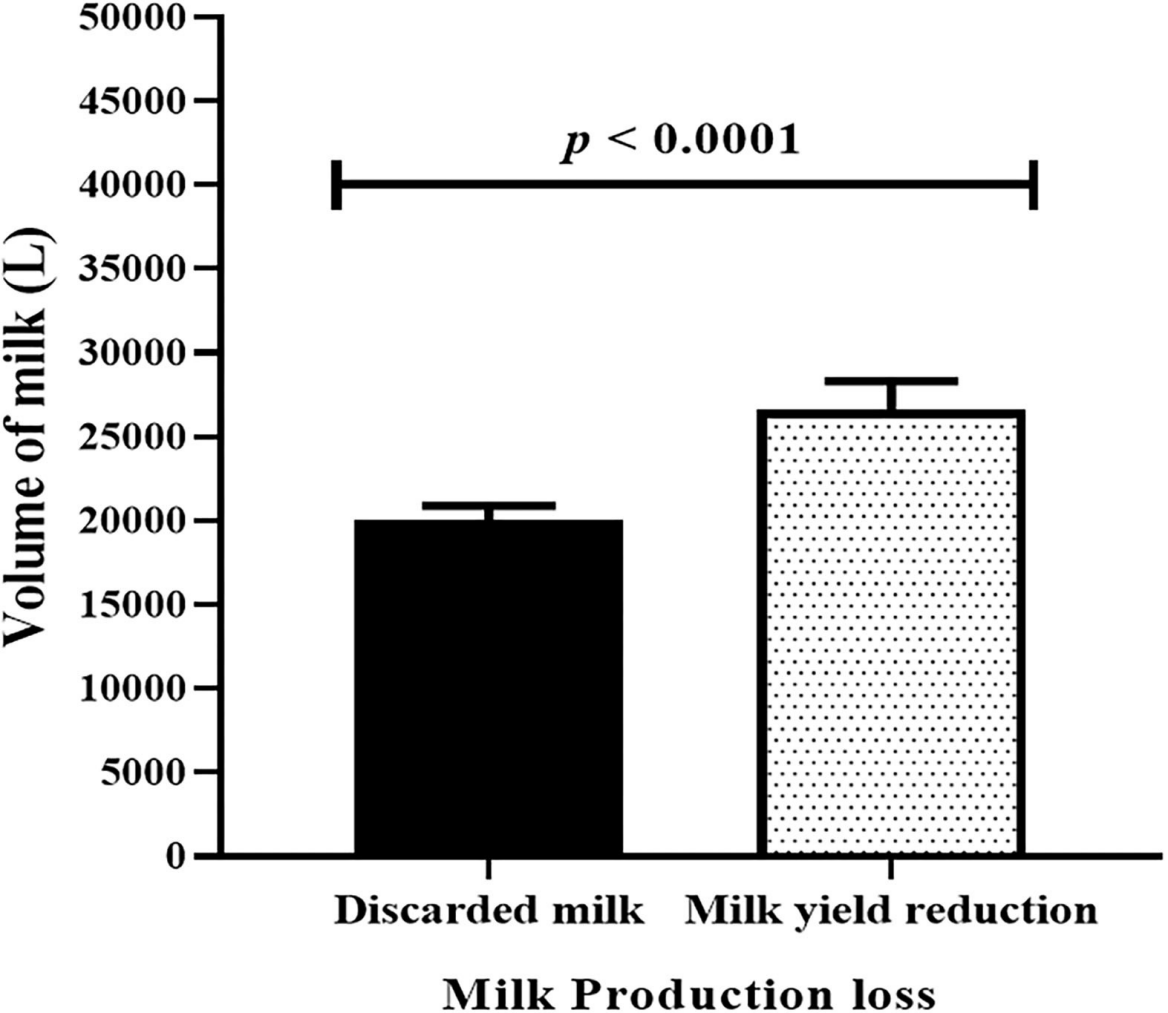
Comparison of monthly discarded milk and monthly reduced milk production due to lactating cows affected by clinical mastitis. Black solid bar error represents mean (±SEM) of monthly discarded milk while dotted solid bar error represents monthly reduced milk production due to clinical mastitis. The monthly reduced milk yield was significantly (paired *t-*test, *t* = 4.8, *p* < 0.0001) higher than monthly discarded milk.

The loss of milk due to clinical mastitis occurs as the result of reduced milk yield and discarded milk. The average monthly milk loss (Mean ± SEM) because of reduction of milk yield due to clinical mastitis in kg was 26,592 ± 1,739 while the average amount monthly discarded milk because of clinical mastitis in kg was 20,014±889. Thus, the average of monthly reduced milk yield in kg was higher than the average of monthly discarded milk by 6,578 ± 1,367 and the difference between the two was statistically significant (paired *t-*test, *t* = 4.8, *p* < 0.0001; Figure 8).

## Discussion

The incidence of bovine clinical mastitis was estimated on 1,300 to 1,482 lactating cows at the National Dairy Farm for the duration of 7 years. The incidence rates were similar during the 6 years of the 7 years while it was high in 2019, which could be associated the high average monthly rainfall and humidity of the 2019. Significant associations were observed between the cumulative average monthly incidence rate of clinical mastitis, and the average monthly rainfall and the average monthly humidity. The highest average cumulative monthly incidence rate was 49 cases per 1,000 cows-year in 2019 while the lowest was 19 cases per 1,000 cows-year in 2021. Previous study conducted in Canada on 106 dairy farms reported an average incidence of 23 cases per 100 cow-years (14), which is significantly higher than that recorded by the present study. Moreover, the incidence rates reported earlier the same authors ranged from 0.7 to 97.4 cases per 100 cow-years. Additional studies conducted in Canada reported 19 and 21.3 cases per 100 cow-years (15, 16), which still significantly higher than the average incidence rate recorded by the present study.

The lower incidence recorded in this study could primarily be due to differences in the climatic conductions of the locations of the farms. All the other studies described above were conducted in the temperate regions contrary to this study that was conducted in the arid region, which is dry and less favorable for the survival of pathogens in the environment. While on the other hand, the wet and rainy climatic conditions of the temperate regions could deteriorate the hygiene of the cows and cow's environment thereby facilitating infection of udder. Previous studies also reported that high producing cows raised in rainy regions are more prone to mastitis due to droplet infection, damp and muddy floors that could facilitate the infection of the udder (17).

The incidences of clinical mastitis were also reported from countries located in tropics and subtropics regions. A longitudinal study was conducted on the incidence of clinical mastitis in Tanzania between July 2003 and March 2005 on 317 lactating cows herded in 87 smallholder dairy herds reported an average incidence rate of 43.3 cases per 100 cow-years (18), which is also higher than that reported by the present study. Furthermore, the result of study conducted in Morocco on 259 cows in 87 different dairy farms recorded a prevalence of 20.5% subclinical mastitis and majority of the cases were caused by environmental pathogens (19). Subclinical mastitis cases are highly likely to progress to clinical mastitis if they are not treated. Furthermore, the result of a study conducted on 1,383 lactating cows in Bangladesh recorded an incidence rate of 43.9 cases per 100 cow-years (20). Additionally, according a review published earlier (21) using 57 studies from across the world, the incidence rate of clinical mastitis ranged from 13 to 40 cases/100 cow years. Furthermore, a pooled prevalence of 12.59% was reported by meta-analysis of 17 studies conducted on clinical mastitis in 6,438 dairy cows in Ethiopia (22), which could also suggest higher incidence of clinical mastitis.

In addition to detection of the abnormalities in milk and udder, the National Dairy Farm monitors the udder health and quality of the milk through conducting the somatic cell count periodically using Saber™ Somatic cell counter. The cut-off value of the Farm is 200,000 cells/mL for classifying bulk tank milk as infected or not. The SCC is being used for monitoring udder health and quality of milk worldwide and different cut-off values are applied to identify infected quarters, cows or herds as reviewed different authors (23, 24). According the review published earlier (23), a cut-off 200,000–250,000 cells/mL of milk is used for classifying udders as infected, which is almost closer to the cut-off value used by the National Dairy Farm. But according a recently published review (24), a cut-off values of 300,000–400,000 cells/mL of milk are used by the European Union, China, New Zealand, Australia, Switzerland, and Canada to classify bulk milk as infected or not. Furthermore, as reviewed by these authors, even higher cut-off values i.e., 500,000 cells/mL and 750,000 cells/mL are being used in South Africa and Brazil, and the USA, respectively. Thus, it is important to follow the trends of SCC at herd level over time, and interfere when the cell counts appear to increase above a given threshold.

The effect season on the incidence clinical mastitis was evaluated in this study. However, contrary to the observations made earlier by other authors (25), the result indicated there was no effect season on the cumulative incidence of clinical mastitis. The possible reason could due to the narrow difference between the hot and the cold seasons of the UAE climate condition. In addition, the barns of National Dairy Farm are equipped with the advanced cooling system that keep the cows cool during the hot season, which further narrowing the difference between the cold and hot seasons at the barn level.

In general, different factors contribute for the differences in the incidence rate of clinical mastitis reported by different studies. The most important factors include the pathogen, host, and environmental factors (26). Host factors such as breed, age, nutritional, and immune status of the dairy cows influence the susceptibility of dairy cows to mastitis; Holstein-Friesian dairy cows more susceptible to mastitis than the other breeds (27, 28). Older cows are more susceptible to mastitis because of their wider teat canal, which facilitates the entrance of bacteria into the udder. Furthermore, lactating cows have higher demand for energy and nutrient for the synthesis of colostrum and milk and thus exhibit negative energy balance that leads to immunosuppression thereby increasing their susceptibility to mastitis (28). Environmental conditions and management practices significantly influence the susceptibility of cows to mastitis. High stocking density, contaminated floor, wet bedding, poor ventilation, and hot and humid climate can promote growth of mastitis pathogens and increased exposure of cows, resulting in higher occurrence of mastitis (29).

In the present study, the milk loss was assessed during the month of the occurrence of the clinical mastitis. Previous longitudinal study indicated that rapid decreasing in milk production during the onset of clinical mastitis (30). Milk losses are due to reduced milk yield and discarded milk. The total monthly milk loss ranged from 2 to 5% of the total milk production in 2021 and 2019, respectively. The result of this study indicated that higher the average monthly incidence rate of clinical mastitis, the greater the average amount of monthly milk production loss. A study conducted on 24,276 Finnish Ayrshire dairy cows reported 1.8–7.4% milk losses due to clinical mastitis (31). Wilson et al. (30) reported the largest decrease in milk production during the first week of the onset of the clinical mastitis. In addition, these authors recorded significant reductions of milk yield during the second, third and fourth weeks post the onset of clinical mastitis. Rajala-Schultz et al. (31) reported that after a cow that contracted mastitis cannot return to the pre-mastitis level.

Although the economic losses associated with the loss of milk due to clinical mastitis was not estimated in the present study, the economic loss could be substantial particularly in 2019. A recent study conducted in Canada indicated that mastitis causes substantial costs (662 Canadian dollar per a milking cow per year) (16). Furthermore, these authors reported that the costs of clinical mastitis are due to culling of cows, reduction of milk yield and discarded milk; each sharing 48, 34, and 11% of the total costs of clinical mastitis. Thus, mastitis is the leading cause of economic loss in dairy industries due to reduced yield and poor quality of produced milk (32). In the present study, the major economic losses were due to reduced milk yield and discarded milk. Particularly, decreased milk production due to the damage to the mammary gland tissues takes a significant share of the total economic losses due to mastitis (33).

Finally, the strength of this study could be due to the analysis of 7 years data on the monthly incidence of clinical mastitis and the associated loss of milk production. The average monthly incidence clinical mastitis and loss of milk production were analyzed for 84 months on the large number of lactating dairy cows. However, this study has limitations as it was conducted on secondary data, which were collected for the purpose of keeping the official records of the Farm.

## Conclusion

Clinical mastitis is considered as the major challenges of the National Dairy Farm although the Farm is doing its maximum efforts in applying preventive measures prior to its occurrence and also treating and effectively managing clinical mastitis cases. The milk loss and associated economic loss were maintained at low level at the National Dairy Farm as compared the milk losses reported from other countries. Mastitis is a complex disease that is influenced by host, environment and pathogen factors. Therefore, the National Dairy Farm should reinforce its mastitis control and preventive measures through identification and treatment of clinical cases, disinfection of teat post-milking, routine maintenance of milking machine, culling of chronic cases, and maintaining of the hygiene of the cows and their barns.

## Data availability statement

The original contributions presented in the study are included in the article/supplementary material, further inquiries can be directed to the corresponding author.

## Ethics statement

Ethical review and approval was not required for the animal study because it was a retrospective data which were collected as part of the routine dairy farm activities. Approval was obtained from the Farm's management to use the data and publish the findings of the analysis. Written informed consent was obtained from the owners for the participation of their animals in this study.

## Author contributions

GA contributed in conceptualizing and leading the study, supporting the data analysis, and drafting and editing of the manuscript. BB and BD contributed in the data analysis, interpretation, and edition of the manuscript. AZ and GK contributed in data collection and data entry. KM, MA, YE, and ME contributed in editing the manuscript. MT contributed in drafting and editing of the manuscript. All authors contributed to the article and approved the submitted version.

## References

[1] Cheng WN Han SG. Bovine mastitis: risk factors, therapeutic strategies, and alternative treatments—a review. Asian Australas J Anim Sci. (2020) 33:1699–713. 10.5713/ajas.20.0156 32777908 PMC7649072

[2] Krishnamoorthy P Goudar AL Suresh KP Roy P. Global and countrywide prevalence of subclinical and clinical mastitis in dairy cattle and buffaloes by systematic review and meta-analysis. Res Vet Sci. (2021) 136:561–86. 10.1016/j.rvsc.2021.04.021 33892366

[3] Bradley AJ. Bovine mastitis: an evolving disease. Vet J. (2002) 164:116–28. 10.1053/tvjl.2002.0724 12359466

[4] Fox LK Kirk JH Britten A. Mycoplasma mastitis: a review of transmission and control. J Vet Med B. (2005) 52:153–60. 10.1111/j.1439-0450.2005.00845.x 16000109

[5] Olde-Riekerink RGM Barkema HW Veenstra S Poole DE Dingwell RT Keefe GP. Prevalence of contagious mastitis pathogens in bulk tank milk in Prince Edward Island. Can Vet J. (2006) 47:567–72. 16808229 PMC1461414

[6] Hogeveen H Huijps K Lam TJGM. Economic aspects of mastitis: New developments. N Z Vet J. (2011) 59:16–23. 10.1080/00480169.2011.547165 21328153

[7] Viguier C Arora S Gilmartin N Welbeck N O'Kennedy R. Mastitis detection: current trends and future perspectives. Trends Biotechnol. (2009) 27:486–93. 10.1016/j.tibtech.2009.05.004 19616330

[8] Bansal BK Gupta DK. Economic analysis of bovine mastitis in India and Punjab —a review. Indian J Dairy Sci. (2009) 62:337–45.

[9] Halasa T Huijps K Østerås O Hogeveen H. Economic effects of bovine mastitis and mastitis management: a review. Vet Q. (2007) 29:18–31. 10.1080/01652176.2007.9695224 17471788

[10] Bradyley AJ Green MJ. Aetiology of clinical mastitis in six somerset dairy herds. Vet Rec. (2001) 146:683–6. 10.1136/vr.148.22.683 11425254

[11] White DG Mcdermott PF. Emergence and transfer of antibiotic resistance. J Dairy Sci. (2001) 84 (E. Suppl.):E151–5. 10.3168/jds.S0022-0302(01)70209-3

[12] UAE Government. National Food Security Strategy 2051. UAE Government (2022). Available online at: https://u.ae/en/about-the-uae/strategies-initiatives-and-awards/federal-governments-strategies-and-plans/national-food-security-strategy-2051 (accessed July 4, 2022).

[13] van Dijk Z. Upward Trend for UAE Dairy Products. Dairy Global (2021). Available online at: https://https//www.dairyglobal.net/industry-and-markets/market-trends/upward-trend-for-uae-dairy-products/ (accessed August 21, 2022).

[14] Olde-Riekerink RG Barkema HW Kelton DF Scholl DT. Incidence rate of clinical mastitis on Canadian dairy farms. J Dairy Sci. (2008) 91:1366–77. 10.3168/jds.2007-0757 18349229

[15] Elghafghuf A Dufour S Reyher K Dohoo I Stryhn H. Survival analysis of clinical mastitis data using a nested frailty cox model fit as a mixed-effects poisson model. Prev Vet Med. (2014) 117:456–68. 10.1016/j.prevetmed.2014.09.013 25449735

[16] Aghamohammadi M Haine D Kelton DF Barkema HW Hogeveen H Keefe GP. Herd-level mastitis associated costs on Canadian dairy farms. Front Vet Sci. (2018) 5:100. 10.3389/fvets.2018.00100 29868620 PMC5961536

[17] Sinha R Sinha B Kumari R Vineeth MR Verma A Gupta ID. Effect of season, stage of lactation, parity and level of milk production on incidence of clinical mastitis in Karan fries and Sahiwal cows. Biol Rhythm Res. (2021) 52:593–602. 10.1080/09291016.2019.1621064

[18] Olivares-Pérez J Kholif AE Rojas-Hernández S Elghandour MMMY Salem AZM Bastida AZ. Prevalence of bovine subclinical mastitis, its etiology and diagnosis of antibiotic resistance of dairy farms in four municipalities of a tropical region of Mexico. Trop Anim Health Prod. (2015) 47:1497–504. 10.1007/s11250-015-0890-8 26255183

[19] Kivaria FM Noordhuizen JPTM Msami HM. Risk factors associated with the incidence rate of clinical mastitis in smallholder dairy cows in the Dar es Salaam region of Tanzania. Vet J. (2007) 173:623–9. 10.1016/j.tvjl.2006.01.009 16516505

[20] Singha S Koop G Persson Y Hossain D Scanlon L Derks M. Incidence, etiology, and risk factors of clinical mastitis in dairy cows under semi-tropical circumstances in Chattogram, Bangladesh. Animals. (2021) 11:2255. 10.3390/ani11082255 34438713 PMC8388477

[21] Jamali H Barkema HW Jacques M Lavallée-Bourget EM Malouin F Saini V. Invited review: incidence, risk factors, and effects of clinical mastitis recurrence in dairy cows. J Dairy Sci. (2018) 101:4729–46. 10.3168/jds.2017-13730 29525302

[22] Girma G Tamir D. Prevalence of bovine mastitis and its associated risk factors among dairy cows in Ethiopia during 2005–2022: a systematic review and meta-analysis. Vet Med Int. (2022) 2022:7775197. 10.1155/2022/7775197 36164492 PMC9509276

[23] Schukken Y Wilson D Welcome F Garrison-Tikofsky L Gonzalez R. Monitoring udder health and milk quality using somatic cell counts. BMC Vet Res. (2003) 34:579–96. 10.1051/vetres:2003028 14556696

[24] Alhussien MN Dang AK. Milk somatic cells, factors influencing their release, future prospects, and practical utility in dairy animals: an overview. Vet World. (2018) 11:562–77. 10.14202/vetworld.2018.562-577 29915493 PMC5993762

[25] Alhussien MN Dang AK. Impact of different seasons on the milk somatic and differential cell counts, milk cortisol and neutrophils functionality of three Indian native breeds of cattle. J Therm Biol. (2018) 78:27–35. 10.1016/j.jtherbio.2018.08.020 30509646

[26] Klaas IC Zadoks RN. An update on environmental mastitis: challenging perceptions. Transbound Emerg Dis. (2018) 65:166–85. 10.1111/tbed.12704 29083115

[27] Washburn SP White SL Green JJT Benson GA. Reproduction, mastitis, and body condition of seasonally calved Holstein and Jersey cows in confinement or pasture systems. J Dairy Sci. (2002) 85:105–11. 10.3168/jds.S0022-0302(02)74058-7 11860102

[28] Shaheen M Tantary H Nabi S. A treatise on bovine mastitis: disease and disease economics, etiological basis, risk factors, impact on human health, therapeutic management, prevention and control strategy. Adv Dairy Res. (2016) 4:150. 10.4172/2329-888X.1000150

[29] Zeinhom MMA Aziz RLA Mohammed AN Bernabucci U. Impact of seasonal conditions on quality and pathogens content of milk in Friesian cows. Asian-Australas J Anim Sci. (2016) 29:1207–13. 10.5713/ajas.16.0143 27165021 PMC4932576

[30] Wilson DJ González RN Hertl J Schulte HF Bennett GJ Schukken YH. Effect of clinical mastitis on the lactation curve: a mixed model estimation using daily milk weights. J Dairy Sci. (2004) 87:2073–84. 10.3168/jds.S0022-0302(04)70025-9 15328219

[31] Rajala-Schultz PJ Grohn YT McCulloch CE Guard CL. Effects of clinical mastitis on milk yield in dairy cows. J Dairy Sci. (1999) 82:1213–20. 10.3168/jds.S0022-0302(99)75344-0 10386307

[32] Gomes F Saavedra MJ Henriques M. Bovine mastitis disease/pathogenicity: evidence of potential role of microbial biofilms. Pathog Dis. (2016) 74:ftw006. 10.1093/femspd/ftw006 26772653

[33] Zhao X Lacasse P. Mammary tissue damage during bovine mastitis: causes and control. J Anim Sci. (2008) 86:57–65. 10.2527/jas.2007-0302 17785603

